# Vitamin Biosynthesis as an Antifungal Target

**DOI:** 10.3390/jof4020072

**Published:** 2018-06-17

**Authors:** Zohar Meir, Nir Osherov

**Affiliations:** Department of Clinical Microbiology and Immunology, Sackler School of Medicine, Tel-Aviv University, Ramat-Aviv, Tel-Aviv 69978, Israel; lightenzm@gmail.com

**Keywords:** antifungals, fungal vitamin metabolism, drug target, essential genes

## Abstract

The large increase in the population of immunosuppressed patients, coupled with the limited efficacy of existing antifungals and rising resistance toward them, have dramatically highlighted the need to develop novel drugs for the treatment of invasive fungal infections. An attractive possibility is the identification of possible drug targets within essential fungal metabolic pathways not shared with humans. Here, we review the vitamin biosynthetic pathways (vitamins A–E, K) as candidates for the development of antifungals. We present a set of ranking criteria that identify the vitamin B2 (riboflavin), B5 (pantothenic acid), and B9 (folate) biosynthesis pathways as being particularly rich in new antifungal targets. We propose that recent scientific advances in the fields of drug design and fungal genomics have developed sufficiently to merit a renewed look at these pathways as promising sources for the development of novel classes of antifungals.

## 1. Introduction

The number of life-threatening fungal infections has risen dramatically over the last twenty years. Recent estimates have identified a global burden of almost two million patients with systemic and invasive fungal infections, including ~700,000 cases of invasive candidiasis, ~500,000 cases of *Pneumocystis jirovecii* pneumonia, ~250,000 cases of invasive aspergillosis, ~220,000 cases of cryptococcal meningitis, and ~100,000 cases of disseminated histoplasmosis [1,2]. The primary reason for this is the rapid rise in the number of chronically immunosuppressed and debilitated patients. This is due to aggressive chemotherapy to treat leukemia and other hematological malignancies, the rise in bone marrow transplantations (BMTs), and AIDS.

Treatments for invasive fungal infections remain unsatisfactory. There are only four classes of established antifungal drugs on the market—polyenes (e.g., amphotericin B formulations), triazoles (e.g., voriconazole), the newly introduced echinocandins (e.g., caspofungin), and allylamines (e.g., terbinafine). Of these, only the first three classes are currently used to treat systemic fungal infections [3]. Nevertheless, despite treatment, there remains an unacceptably high mortality rate in high-risk patients. In addition, some of the current antifungal treatments interact unfavorably with other medications, have resistance problems, a low spectrum of activity, limited formulation, are fungistatic as opposed to fungicidal, and are frequently toxic [3]. This is primarily because fungi are eukaryotes and share many biochemical pathways and subcellular structures with mammalian cells. Consequently, most currently used antifungals are not truly fungal-specific. Only the echinocandins inhibit a specific target of the fungal cell-wall, and indeed exhibit an excellent safety profile and clinical efficacy [4]. However, they are not orally available, have a narrow therapeutic range, and are fungistatic against molds [4].

Because of downsizing, consolidation, and low profitability, most large pharmaceutical companies have considerably reduced or even halted their efforts to develop novel antifungals, even as resistance to the existing drugs rapidly emerges amongst clinical isolates [5]. Thus, there is an urgent and unmet need to develop additional and novel antifungal drugs that inhibit essential fungal-specific cellular targets and pathways [6].

During the last two decades, extensive molecular studies have helped identify several fungal-specific drug targets shared by the most important human pathogenic fungi, *Candida albicans*, *Aspergillus fumigatus,* and *Cryptococcus neoformans*, and not found in higher eukaryotes including humans [7,8,9,10]. They include essential genes that synthesize, maintain, and control the structure of the fungal cell wall (e.g., *FKS1* glucan synthase, *PKC* protein kinase C, *CHS1* chitin synthase) [11], unique pathways participating in the uptake of iron (e.g., *SIT1* siderophore transporter, *sidA* siderophore biosynthesis, *FTR1* iron permease) [12,13], zinc and copper (e.g., *zrfA-C* zinc transporters, *crpA* copper transporter) [14], and the transport and synthesis of essential aromatic amino acids, metabolic precursors, and vitamins (e.g., *ARO1-9*, *HisB*, *SNO1/SNZ1*, *ABZ1-2*, and *PabaA*, respectively) [15,16,17,18]. Importantly, deletion of genes participating in these pathways, in one or more of the main systemic human fungal pathogens (*C. albicans*, *A. fumigatus,* and *C. neoformans*), abolishes their virulence [9,19,20,21,22,23], suggesting that they are promising drug targets.

This review focuses exclusively on the essential vitamin biosynthetic pathways (A, B1–12, C, D, E, and K) and their suitability as antifungal drug targets. Among the criteria qualifying them as valid drug targets are their absence in the human host, conservation among fungi, well-understood biochemistry/structural data, and essentiality for fungal growth in the infected host. An important caveat is that the host niche should not supply the infecting fungus with those nutrients, as it would allow it to bypass pathway inhibition. A notable limitation in our analysis is that the virulence of the fungal vitamin auxotrophs described here was tested in diverse infection models, using different mouse strains, immunosuppressive regimens (neutropenic, non-neutropenic), infection routes (disseminated, lungs), and readouts (mortality, fungal load) that can result in different outcomes. Thus, our approach has been to focus on pathways in which auxotrophies result in avirulence in the maximal number of infectious models, giving the most robust results.

There are several overlooked advantages in targeting fungal-unique enzymes participating in vitamin biosynthesis: (i) The substrates and products of these enzymes are well-characterized small molecules with high potential druggability. This has already been exploited in the generation of inhibitors of vitamin B5 (pantothenic acid) and B9 (folate) biosynthesis (see relevant sections below) [11]; (ii) competitive inhibitors of vitamin biosynthesis can be identified relatively easily because their activity is blocked upon addition of excess product. This property can be exploited to rapidly identify pathway-specific inhibitors from large compound libraries [24,25]. Note that this approach can effectively identify metabolic inhibitors in general (e.g., essential amino acids, nucleotides, metal uptake, etc.); (iii) several of the vitamin biosynthetic pathways, including vitamin B2 (riboflavin), vitamin B5 (pantothenic acid), and vitamin B9 (folate) biosynthesis, supply cofactors for hundreds of enzymes involved in essential metabolic processes. Therefore, inhibition of these vitamin biosynthetic pathways will have a very large inhibitory cascade effect on many metabolic processes, potentially leading to lethal cellular damage; (iv) some of the vitamin pathways supply precursors needed for the biosynthesis of known virulence factors. As a result, inhibiting these pathways will also inhibit the production of the dependent virulence factors. For example, riboflavin/vitamin B2 serves as a cofactor for ornithine-N^5^-monooxygenase SidA, which catalyses the initial step in siderophore biosynthesis in *A. fumigatus*. Siderophores are essential virulence factors, allowing the fungus to overcome severe iron limitation in the host [26]. Inhibition of riboflavin biosynthesis strongly (>80%) reduces the production of siderophores [27], delivering an unexpected advantage by inhibiting fungal iron acquisition and growth during infection.

Despite their potential, to date there are few antifungals targeting the vitamin biosynthetic pathways. Pneumocystis pneumonia, caused by the yeast *P. jirovecii*, is treated with a synergistic combination of sulfamethoxazole and trimethoprim, which inhibit two key vitamin B9/folate biosynthetic enzymes. A similar combinatorial approach is used to treat malaria caused by *Plasmodium falciparum,* as well as many types of bacterial infections (see below).

While we have limited our target search to include only fungal-specific enzymes not found in humans, there are many examples of existing antimicrobial drugs (e.g., azole and allylamine antifungals, antimicrobial DHFR inhibitors, etc.) and pipeline antifungals (AX001 inositol acyltransferase inhibitor, F901318 dihydroorotate dehydrogenase inhibitor) that inhibit targets shared with humans [6]. The antifungal specificity of these drugs was achieved by painstakingly optimizing their structure to bind more tightly and selectively to the microbial enzyme.

## 2. The Vitamin A, C, D, E, and K Pathways Are Not Suitable as Antifungal Targets

The first part of this review briefly describes the essential vitamin biosynthetic pathways that are unsuitable, in our opinion, for the development of new antifungals. They include the vitamin A, C, D, E, and K pathways.

Vitamin A compounds (retinol, retinal, retinoic acid, and their precursors, the carotenoids) are important for growth and development, for the maintenance of the immune system and good vision [28]. Animals lack the vitamin A biosynthetic pathway and rely on exogenous sources such as plants. Carotenoids are organic pigments that are found in the chloroplasts and chromoplasts of plants and some other photosynthetic organisms, including some bacteria and fungi. The most common carotenoids include lycopene and the vitamin A precursor β-carotene. β-carotene is an intense red-orange pigment abundant in plants and fruits. Fungi produce carotenoids for different non-essential functions, including stress tolerance and synthesis of physiologically active by-products. However, in human pathogenic fungi that produce carotenoids (*Rhizopus* and *Aspergillus* spp.), mutants unable to produce them do not display phenotypic alterations in the laboratory, apart from lack of pigmentation [29]. Many fungi do not produce carotenoids, including pathogenic species of *Candida* and *Cryptococcus* [29]. Thus, the carotenoid pathway is not a good target for the development of antifungals.

Vitamin C or ascorbic acid is a cofactor for a number of enzymes and an important antioxidant. It is produced in all higher plants and most animals. Humans and anthropoid apes cannot synthesize ascorbate, because of mutations in the l-gulono-γ-lactone oxidase (*GLO*) gene catalyzing the last step in its biosynthesis [30]. In the pathogenic fungus *C. albicans*, deletion of the *GLO* homolog *alo1* increases sensitivity to oxidative stress in vitro and attenuates virulence in infected immunocompetent mice [31]. Nevertheless, deletion of the *A. fumigatus GLO* homolog *Afu1g14950* does not affect oxidative stress sensitivity or virulence (our unpublished results). Collectively, these results suggest that even the most potent inhibitor of Alo1 would only partially reduce *C. albicans* virulence and would probably be ineffective against *A. fumigatus*.

Finally, vitamin D (vitamin D_2_/ergocalciferol) is produced by fungi from ergosterol in a non-enzymatic photochemical reaction catalyzed by UV-B rays [32]. It thus lacks a druggable target, while vitamins E and K are only synthesized by plants and not fungi.

## 3. The B vitamins as Antifungal Targets

B vitamins are a chemically diverse group of water-soluble compounds that are important cofactors in cell metabolism. They include vitamins B1 (thiamine), B2 (riboflavin), B3 (niacin), B5 (pantothenic acid), B6 (pyridoxine), B7 (biotin), B9 (folate), and B12 (cobalamin). We will briefly describe their biosynthetic pathways and focus on those containing targets suitable for the development of new antifungals. A summary is provided in Table 1.

### 3.1. Vitamin B1 (Thiamine)

**Figure 1:**
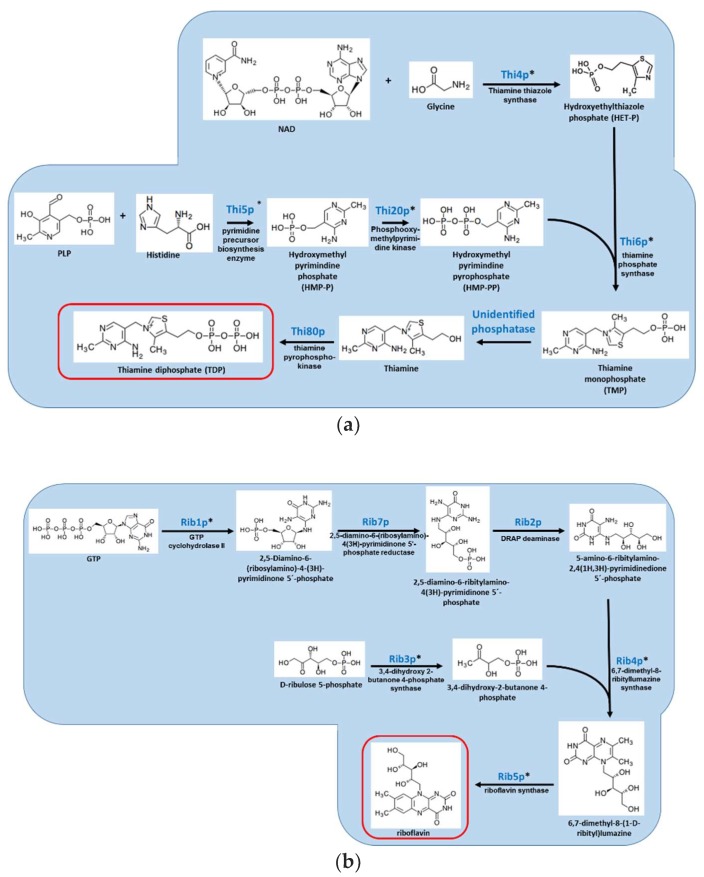

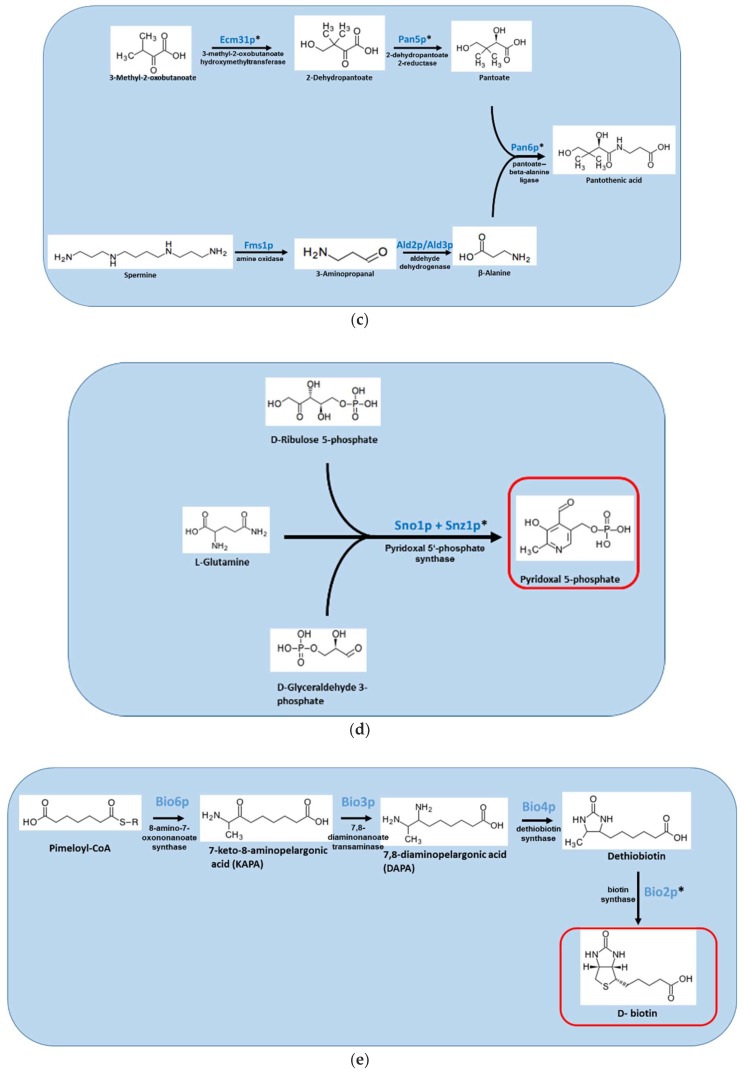

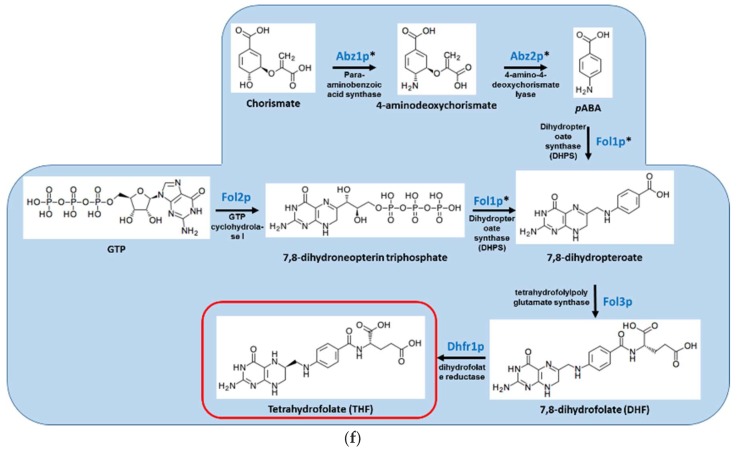
Overview of the *S. cerevisiae* pathways responsible for the biosynthesis of (**A**) vitamin B1/thiamine; (**B**) vitamin B2/riboflavin; (**C**) vitamin B5/pantothenic acid; (**D**) vitamin B6/pyridoxine; (**E**) vitamin B7/biotin; and (**F**) vitamin B9/folate. Red box indicates the vitamin product. Genes denoted with * have no human homologs.

Thiamine is synthesized by bacteria, plants, and fungi but not by animals. It is a cofactor in carbohydrate and amino acid metabolism, glycolysis, TCA cycle, and the pentose shunt [50]. Thiamine is generated by the Thi6p-dependant coupling of thiazole and pyrimidine, each synthesized by a separate pathway (Figure 1A). Fungi can also obtain thiamine from their environment, using a dedicated transporter, Thi7p. The importance of fungal thiamine biosynthesis during animal infection has only been determined in the *Aspergilli*. In *Aspergillus nidulans*, a thiamine auxotroph generated by point mutation of *thi4* was fully virulent in a murine model of systemic infection [33]. In *A. fumigatus*, a thiamine auxotroph generated by *thi6/thiB* gene deletion showed attenuated virulence in both pulmonary and systemic models of infection. There was no difference between wild-type and mutant in fungal load and histopathology, suggesting that there is sufficient thiamine in the infected host to allow strong growth of the *thi6/thiB* mutant [27]. Therefore, inhibiting thiamine biosynthesis may not be a good strategy for antifungal development, unless perhaps if combined with the development of inhibitors of thiamine uptake. Interestingly, thiamine biosynthesis is essential for virulence in the plant pathogenic fungus *Verticillium dahlia*, suggesting that this pathway could be suitable for the development of agricultural fungicides [51].

### 3.2. Vitamin B2 (Riboflavin)

Riboflavin in its active forms, flavin adenine dinucleotide (FAD) and flavin mononucleotide (FMN), functions as a cofactor for numerous flavocoenzyme-catalyzed reactions, including electron transport, fatty acid oxidation, and vitamin B3/B6/B9 synthesis. Riboflavin is synthesized from one molecule of GTP and two molecules of ribulose 5 phosphate by six enzymes (Rib1–Rib5p and Rib7p) [52] (Figure 1B). In *S. cerevisiae*, deletion of *RIB1*-*RIB5* results in riboflavin auxotrophy.

The virulence of riboflavin auxotrophs has been assessed in the pathogenic fungi *C. albicans* [9], *Histoplasma capsulatum* [38], *A. nidulans* [33], and *A. fumigatus* [27]. In *C. albicans*, conditional repression of Rib2 results in avirulence in systemically infected immunocompetent mice [9]. In *H. capsulatum*, disruption of Rib2 prevents fungal proliferation inside macrophages in vitro. Virulence in intranasally infected immunocompetent mice is severely attenuated as evidenced by an inability to replicate in the lungs or disseminate [38]. Rib2 may be unsuitable as a drug target because it has high homology to an uncharacterized pseudouridylate synthase domain-containing protein (NP 689473) in humans. Mutational inactivation of Rib1 in *A. nidulans* attenuates virulence in systemically infected immunocompetent mice [33]. Deletion of Rib1 (*riboB*) in *A. fumigatus* abolishes virulence in intranasally infected cortisone-compromised mice and systemically infected neutropenic mice, and attenuates virulence in intranasally neutropenic mice [27]. Under riboflavin limitation, the Δ*riboB* strain is more sensitive to nitric oxide and produces less siderophores, further reducing its ability to survive in the host [27]. Together, these findings indicate that riboflavin biosynthesis offers attractive targets for the development of novel antifungals. The crystal structure of the last two enzymes in the pathway, Rib4 lumazine synthase and Rib5 riboflavin synthase, has been elucidated in the yeast *Schizosaccharomyces pombe*, *C. albicans,* and *Candida glabrata* [40]. Small-molecule screens have identified several compounds that inhibit the activity of purified *S. pombe* lumazine synthase Rib4p, although they lack antifungal activity because of poor cell penetration [42]. To identify compounds that inhibit riboflavin biosynthesis and can enter the fungus, we screened a small-molecule library against *A. fumigatus* and identified two compounds that lose their antifungal activity when excess riboflavin is added to the growth medium, bypassing the need for *de novo* synthesis [24]. We presume that these compounds inhibit a key step in fungal riboflavin biosynthesis. Ongoing work will determine their precise targets.

Interestingly, many species of bacteria, including *Mycobacterium tuberculosis*, lack exogenous riboflavin uptake systems and are completely reliant on endogenous biosynthesis. Several substrate analogs or antimetabolites with moderate activity against purified Rib4p or Rib5p and weak activity against *M. tuberculosis* bacteria in culture have been described. Taken together, these findings demonstrate the feasibility of developing riboflavin biosynthesis inhibitors as antimicrobial drugs [53,54].

### 3.3. Vitamin B3 (Niacin/nicotinic Acid)

Vitamin B3 or niacin is a precursor of NAD and NADP, which are coenzymes of many dehydrogenases. Three lines of evidence suggest that the niacin biosynthetic pathway is not a good target for antifungal development: (i) Niacin is not strictly an essential vitamin, as it can be synthesized *de novo* from tryptophan (through the conserved kynurenine pathway) by most organisms, including plants, bacteria, fungi, and animals [55]; (ii) niacin auxotrophy does not affect virulence in *A. nidulans* [33]; and (iii) *C. glabrata*, which is a natural niacin auxotroph, is nevertheless an effective pathogen [56]. 

### 3.4. Vitamin B5 (Pantothenic Acid)

Pantothenic acid is a precursor of the essential cofactor CoA, which functions in many central cellular metabolic pathways, including fatty acid and carbohydrate metabolism, as well as polyketide and nonribosomal peptide biosynthesis [57]. Four enzymes, encoded by the genes *panB-E*, are responsible for the biosynthesis of pantothenic acid from aspartate/spermine and ketoisovalerate in a pathway found in most bacteria, fungi, and plants, but not in mammals (Figure 1C). In all organisms, exogenously available pantothenic acid can be imported into the cell by a pantothenate transporter. In *S. cerevisiae*, deletion of *ECM31* and *PAN6*, homologous to bacterial *panB* and *panC*, results in pantothenic acid auxotrophy. In *C. albicans*, downregulation of *ECM31* expression strongly reduces fungal burden [9]. In the pathogenic yeast *H. capsulatum*, auxotropic *pan6* RNAi knockdowns show reduced lung and spleen fungal loads in infected mice, indicating reduced virulence [38]. In *A. nidulans*, deletion of *pantoA/pan6* and *pantoB/ECM31* results in pantothenic acid auxotrophy [58]. In *A. fumigatus, panA/pan6* deletion also results in pantothenic acid auxotrophy. In vitro, growth of this mutant under limiting pantothenic acid and iron is strongly reduced, probably because pantothenic acid is needed for siderophore biosynthesis. The *A. fumigatus Δpan6* mutant is avirulent in systemically infected neutropenic mice and strongly reduced in virulence in lung-infected neutropenic mice [27]. Importantly, pantothenic acid biosynthesis has been studied as a possible antibacterial target since the 1940s. The enzymes encoding *panB* were purified and crystalized in *E. coli* and *M. tuberculosis*, enabling accurate determination of their structure and the development of specific inhibitors [57]. A major effort has been made to develop pantothenate synthase (*panC*) inhibitors active against *M. tuberculosis*, as this enzyme is essential for virulence [59]. While these approaches yielded effective inhibitors (submicromolar range) of the purified enzyme, their activity was strongly reduced (~two orders of magnitude) against the intact organism and none was tested in vivo. It would be of great interest to elucidate the crystal structure of a fungal pantothenate synthase and to test the activity of available bacterial inhibitors and specifically designed inhibitors against the purified fungal enzyme and the intact fungus in the presence/absence of exogenous pantothenic acid.

### 3.5. Vitamin B6 (Pyridoxine)

Vitamin B6, in the form of pyridoxal 5′-phosphate (PLP), is a cofactor for a large number of essential enzymes taking part in carbohydrate, amino acid, and fatty acid metabolism. PLP can also act directly as a protective agent against reactive oxygen species [60]. Bacteria, fungi, and plants are able to synthesize vitamin B6, whereas most animals, including humans, lack this ability and acquire it from their food. Fungi and plants use the deoxy-xylose 5′-phosphate (DXP)-independent vitamin B6 biosynthesis pathway to generate PLP *de novo*. Two synthase proteins, encoded by the *SNZ1* and *SNO1* genes in *S. cerevisiae*, directly synthesize PLP from ribose 5′-phosphate or ribulose 5′-phosphate, in combination with glyceraldehyde 3′-phosphate and glutamine (Figure 1D) [61]. Baker’s yeast also use the high-affinity transporter Tpn1p to uptake pyridoxine from their environment [62]. Among the pathogenic fungi, pyridoxine auxotrophs have only been generated in *A. nidulans* and *A. fumigatus*. In *A. nidulans*, mutational inactivation of *pyroA*, the homolog of *S. cerevisiae SNZ1*, results in auxotrophy and strongly attenuates virulence in systemically infected mice [33]. Similarly, deletion of *A. fumigatus pyroA* results in auxotrophy and strongly attenuates virulence (70% survival) in neutropenic lung-infected mice, although virulence is only partially attenuated in systemically infected neutropenic mice (100% mortality 10 days post-infection) [27]. Therefore, inhibiting PLP biosynthesis may not be an optimal antifungal strategy unless pyridoxine uptake is also blocked. Interestingly, deletion of *PDX1*, the homolog of *S. cerevisiae SNZ1* in the pathogenic bacteria *M. tuberculosis* and *Helicobacter pylori,* results in pyridoxine auxotrophy and severely attenuates colonization in mice, suggesting that this pathway contains potent antibacterial drug targets [63,64]. The *pdx1* gene of the malaria parasite *P. falciparum* is also essential for virulence, and inhibitors with activity against intact plasmodium have been described [65,66].

### 3.6. Vitamin B7 (Biotin)

Biotin, a prosthetic group in carboxylases/decarboxylases, is produced by plants, bacteria, and most fungi. In fungi, biotin is sequentially synthesized from pimeloyl-CoA by three Bio enzymes: Bio6p, a KAPA synthase; Bio3/4p, a chimeric protein composed of DTB and DAPA synthases; and Bio2p, a biotin synthase (Figure 1E) [67]. Several lines of evidence suggest that the biotin biosynthetic pathway is not suitable as an antifungal target: *C. albicans* lacks *BIO6* and is a natural biotin auxotroph in vitro. Despite this, it is a successful pathogen and grows well in vivo [45]. Likewise, RNAi knockdown/mutation/gene deletion of *bio2/bioB* in *H. capsulatum*, *A. nidulans,* and *A. fumigatus*, respectively, results in biotin auxotrophy in vitro, but does not affect virulence in infected animals, suggesting there is sufficient biotin in the host to allow normal fungal growth and virulence per [33,38] and our unpublished findings.

### 3.7. Vitamin B9 (Folate)

Folates are essential cofactors for the biosynthesis of purines and thymidylate, and hence for DNA replication. Bacteria, plants, and fungi can synthesize folate *de novo*, generating dihydrofolate (DHF) from para-aminobenzoic acid (PABA; derived from chorismate and glutamine) and dihydropteridine diphosphate (DHPP; derived from GTP). This reaction is sequentially catalyzed by the enzymes dihydropteroate synthase (DHPS) encoded by *FOL1* and dihydrofolate synthase (DHFS) encoded by *FOL3* (Figure 1F). Humans lack those enzymes and must obtain folate in the form of DHF from their food, subsequently converting it into tetrahydrofolate (THF) in a reaction catalyzed by dihydrofolate reductase (DHFR). DHFR is highly conserved among all animals, plants, and microorganisms. In *S. cerevisiae*, deletion of *ABZ1*, encoding PABA synthase, and *FOL1/DHPS* result in PABA and folate auxotrophy, respectively [68]. The importance of the pathway in fungal virulence has only been studied in *A. nidulans* and *A. fumigatus*. In both fungi, deletion of *pabA/ABZ1*, encoding PABA synthase, results in PABA auxotrophy and total avirulence [19,46]. Virulence of the *pabA* null mutant was restored when the mice received PABA in their drinking water. Importantly, withdrawal of PABA supplementation during late infection blocked further lethality, suggesting that inhibitors of *pabA* will likely be active against established infection. Inhibitors of bacterial PABA synthase were recently identified but have not undergone in vivo or antifungal testing [69]. *FOL1/DHPS* is also a viable target for antifungal development: Deletion of *A. fumigatus FOL1/DHPS* (*Afu2g09840*) is lethal and cannot be complemented by folate or folinic acid supplementation, suggesting that the fungus relies exclusively on endogenous production (our unpublished findings). Inhibitors of bacterial DHPS, better known as sulfa drugs, have been in clinical use since the 1930s [70]. They are similar to the substrate PABA and competitively inhibit its binding to DHPS. They also react with the second substrate, DHPP, depleting its pool and forming an inhibitory dead-end product. Current sulfa drugs exhibit only weak antifungal activity. Derivatives with higher activity against purified *C. albicans* DHPS were identified but they are ineffective against the whole organism, possibly because of efflux [49]. The *S. cerevisiae* DHPS crystal structure is available and could be used to facilitate the discovery of improved fungal DHPS inhibitors. An attractive option is to develop bisubstrate inhibitors mimicking both PABA and DHPP. This can increase their affinity and specificity compared with the existing sulfa monosubstrate inhibitors [48].

The enzyme DHFR is highly conserved between humans and microorganisms, and seems an unlikely candidate for the development of antimicrobials. Nevertheless, drugs that selectively inhibit human DHFR (e.g., the chemotherapy agent methotrexate) and microbial DHFR (e.g., trimethoprim) were developed and are in widespread clinical use [16]. They are competitive inhibitors that mimic the natural substrate, DHF. However, for both antimicrobial DHPS and DHFR drugs, resistance rapidly emerges because of point mutations in the active site of the enzyme. This problem has been addressed by combination therapy. The simultaneous inhibition of DHPS and DHFR is both synergistic and highly effective in combatting resistance. It is used to treat bacterial infections (e.g., resprim, containing sulfamethoxazole and trimethoprim), malaria (e.g., Lapdab, a combination of chlorproguanil and dapsone), and fungal pneumonia caused by *P. jorovecii* (resprim) [71].

### 3.8. Vitamin B12 (Cobalamins)

Cobalamins are organometallic cofactors used by a limited number of essential enzymes, notably methionine synthase and methylmalonyl-CoA mutase [72]. Only bacteria and archaea have the enzymes required for vitamin B_12_ synthesis. Fungi, plants, and animals (including humans) are incapable of producing vitamin B_12_ and thus this pathway does not constitute a viable antifungal target.

## 4. Metabolic Pathway Inhibitors and the Generation of Resistance

Experience has taught us that antimicrobials targeting metabolic pathways are highly prone to the development of resistance (e.g., antifolates, azoles) because of mutations in the target enzyme. Nonetheless, many strategies can be used to slow down the development of drug resistance. They include: (i) The use of drug combinations (e.g., inhibiting both DHPS and DHFR activity in folate biosynthesis–see above); (ii) generating drugs with multiple targets; (iii) designing drugs that produce toxic end products or metabolites (as occurs with azoles) and which are fungicidal rather than fungistatic, giving no time for resistance to develop (e.g., amphotericin B); (iv) developing drugs targeting conserved substrates or products rather than easily mutable enzymes (like vancomycin and amphotericin B); (v) more precise targeting of the drug (e.g., localizing azoles to their target enzyme in the ER [73]); and (vi) judicious use of new drugs, including limiting their use to humans.

## 5. Conclusions

In summary, the vitamin B2 (riboflavin), B3 (pantothenic acid), and B9 (folate) pathways appear to offer the most attractive antifungal drug targets among the essential vitamin biosynthetic pathways. They contain well-characterized enzymes that are essential for fungal virulence, including one pathway, folate biosynthesis, which has already proved its worth as an antimicrobial and antifungal drug target. Scientific knowledge in the field of drug design and fungal genomics has developed sufficiently to merit a renewed look at metabolic antifungals: Diverse compound libraries or fragment-based drug discovery can be used to identify promising hit compounds [74]. Whole-cell screens coupled with competition assays can rapidly identify pathway-specific inhibitors of vitamin biosynthesis. Whole genome sequencing (WGS)-based genetic analyses and deletion library screens can identify the mode of action of active compounds in diverse pathogenic fungi. Computer-based drug design using crystallographic data of the target enzyme interacting with the initial screen hits can quickly generate templates for increasingly potent and selective inhibitors. These rational multifaceted modern approaches should also incorporate innovations (fungicidal, generating toxic metabolites, having several targets, etc.) that slow or halt the development of resistance. Together, a concerted worldwide effort can generate new classes of highly effective anti-metabolic drugs for the treatment of increasingly resistant and widespread systemic fungal infections.

## Figures and Tables

**Table 1:** Evaluation of the suitability of fungal vitamin B biosynthetic genes for antifungal development.

Pathway	Gene *	Essential for Fungal Virulence	FUNGAL CRYSTAL STRUCTURE	Fungal Inhibitors Developed
**Vitamin B1 thiamine**	*THI4* *THI5* *THI6* *THI20*	No [33] ND ^†^ No [27] ND	Yes [34] Yes [35] Yes [36] Yes [37]	No No No No
**Vitamin B2 riboflavin**	*RIB1* *RIB2* *RIB3* *RIB4* *RIB5*	Yes [27,33] Yes [9,38] ND ND ND	No No Yes [39] Yes [40] Yes [41]	No No No Yes [42] ^‡^ No
**Vitamin B5 pantothenate**	*ECM31* *PAN2* *PAN5* *PAN6*	Yes [9] ND ND Yes [27,38]	No Yes [43] No No	No No No No
**Vitamin B6 pyridoxine**	*SNZ1*	Partially [27,33]	Yes [44]	No
**Vitamin B7 biotin**	*BIO2*	No [33,38,45]	No	No
**Vitamin B9 folate**	*ABZ1* *ABZ2* *FOL1*	Yes [19,46] ND Essential gene	No Yes [47] Yes [48]	No No Yes [49] ^‡^

* *Saccharomyces cerevisiae*; ^†^ ND = not determined; ^‡^ Only active against recombinant fungal enzyme.
